# Fatal incomplete Kawasaki disease in a six-month-old infant from Ethiopia: a case report: Author

**DOI:** 10.1186/s12887-026-06753-2

**Published:** 2026-03-19

**Authors:** Yadiel Tegene Hailegiorgies

**Affiliations:** Department of Pediatrics, Soddo Christian Hospital, Soddo, Ethiopia

**Keywords:** Kawasaki disease, Incomplete kawasaki disease, Coronary artery aneurysm, Cardiogenic shock, Infant, Ethiopia, Case report

## Abstract

**Background:**

Kawasaki disease (KD) is an acute systemic vasculitis of childhood and the leading cause of acquired heart disease in children in developed countries. Infants frequently present with incomplete disease, resulting in delayed diagnosis and increased risk of coronary complications. Data on KD from Africa, particularly Ethiopia, remain extremely limited. This is the first reported case of incomplete KD in an Ethiopian infant.

**Case presentation:**

A fatal case of a six-month-old Ethiopian female infant with missed incomplete Kawasaki disease is reported. At four months of age, she presented with nine days of unexplained fever and markedly elevated inflammatory markers but lacked the classic clinical features of KD. The diagnosis was not considered and she was empirically treated for presumed infectious causes. Two months later she presented in cardiogenic shock. Echocardiography revealed giant coronary aneurysms with intraluminal thrombosis and severely depressed left ventricular systolic function. Despite intensive care support, the patient died within 12 hours of admission.

**Conclusion:**

This case highlights the devastating consequences of missed and delayed recognition of incomplete KD in young infants. Clinicians must maintain a high index of suspicion for KD in infants with prolonged unexplained fever and elevated inflammatory markers, even in the absence of classic clinical features. Strict adherence to established diagnostic algorithms is essential to prevent catastrophic cardiac complications, particularly in low-resource settings where awareness of KD remains limited.

**Supplementary Information:**

The online version contains supplementary material available at 10.1186/s12887-026-06753-2.

## Background

Kawasaki disease (KD) is an acute febrile systemic vasculitis of unknown etiology that predominantly affects children under five years of age and represents the leading cause of acquired heart disease in children in developed countries. [1] Diagnosis is clinical, as no pathognomonic diagnostic test exists. Classic KD is defined by persistent fever together with at least four of five principal clinical features: polymorphous rash, bilateral non-exudative conjunctival injection, oral mucosal changes, cervical lymphadenopathy, and extremity changes [2].

Although complete KD has been described in infants as young as six weeks of age [3], infants younger than one year commonly present with incomplete or atypical KD, with fewer classical manifestations. This often leads to delayed diagnosis and treatment, placing infants at substantially higher risk of developing coronary artery aneurysms [4].

Published data on KD from Africa remain scarce. Most available reports consist of isolated case reports from a small number of East African countries, and epidemiologic data are lacking [5–7]. To the best of the author’s knowledge, only one case report from Ethiopia has previously been published, describing an adult with a giant coronary artery aneurysm presumed to be secondary to remote KD [8].

This is the first reported case of incomplete KD in an Ethiopian infant, highlighting the diagnostic challenges and potentially catastrophic consequences of delayed recognition in resource-limited settings.

### Case presentation

A six-month-old Ethiopian female infant presented to the emergency department with a two-day history of grunting, fast breathing and non-bilious vomiting. Her parents reported episodes of apparent abdominal pain, accompanied by excessive crying and irritability.

She had been evaluated in the emergency department two days earlier. Initial investigations included a complete blood count (CBC), urinalysis, and stool examination. The CBC showed leukocytosis with a white blood cell count (WBC) of 13.7 × 10^9^/L, lymphocyte predominance (64.2%), moderate anemia (hemoglobin 9.5 g/dL) and platelet count of 576 × 10^9^/L. Urinalysis and stool exam were unremarkable. She was diagnosed with presumed community-acquired pneumonia, prescribed azithromycin syrup, and discharged home.

Her symptoms worsened, prompting re-presentation to the emergency department with increased respiratory distress, persistent vomiting, and continued grunting. An abdominal ultrasound was performed to evaluate for possible intussusception. Intussusception was excluded but the ultrasound revealed free peritoneal fluid, bilateral large pleural effusions and markedly dilated cardiac chambers.

Her clinical condition deteriorated rapidly, with worsening hypoxia and increased work of breathing, necessitating transfer to the intensive care unit (ICU), where she was commenced on bilevel positive airway pressure (BiPAP) support.

On admission to the ICU, she was critically ill. Vital signs revealed a heart rate of 172 beats per minute, respiratory rate of 43 breaths per minute, temperature of 34.1 °C, and hypotension with blood pressure of 48/29 mmHg. Her weight was 7.8 kg and length was 68 cm.

**Fig. 1:**
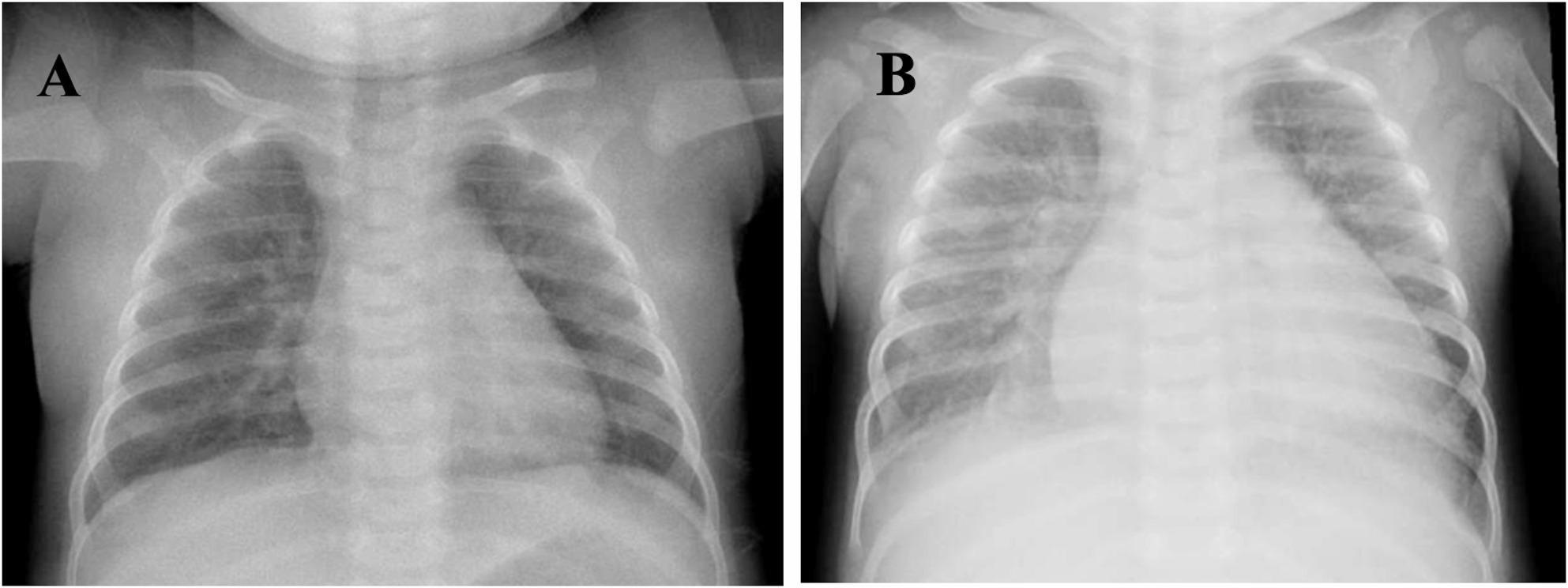
Serial chest radiographs demonstrating progression to severe cardiomegaly. **A **Chest radiograph obtained at four months of age during initial hospitalization demonstrating a normal cardiac silhouette. **B **Chest radiograph obtained at the final presentation at six months of age demonstrating marked cardiomegaly (cardiothoracic ratio 0.69) with pulmonary edema

**Fig. 2:**
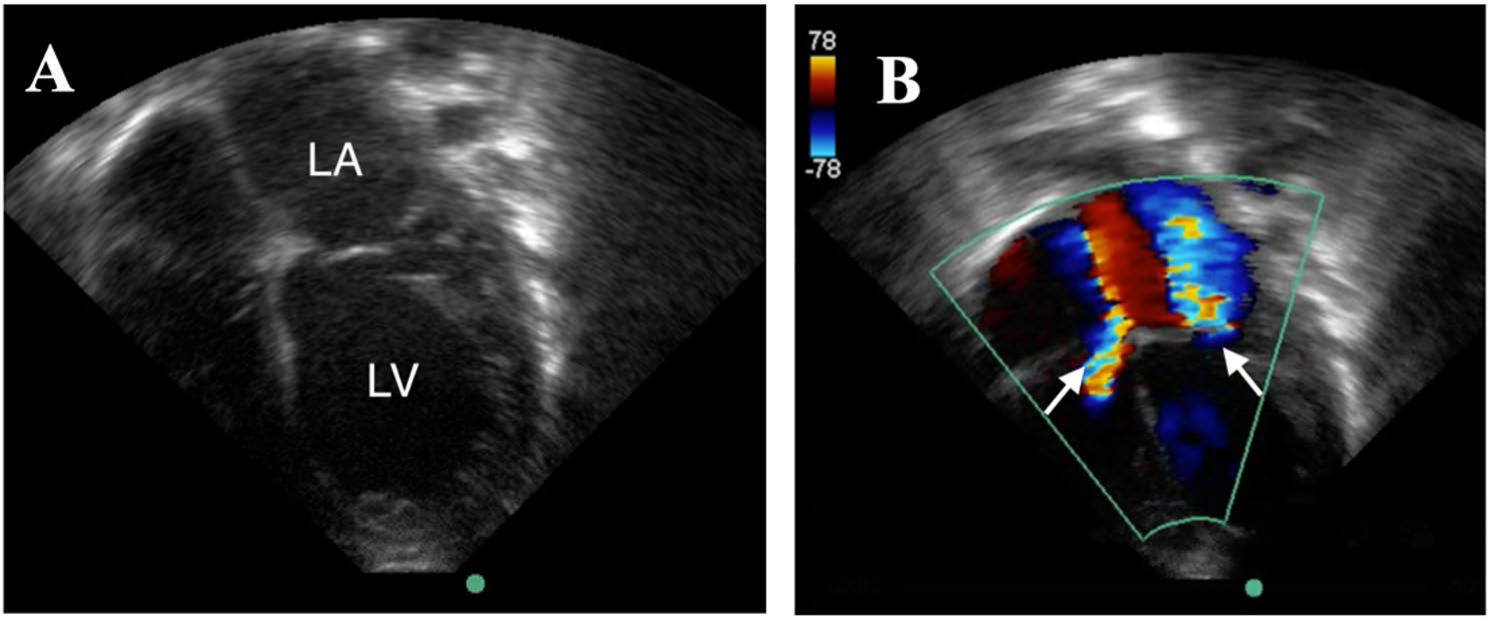
Transthoracic echocardiography demonstrating apical four-chamber view. **A **Apical four-chamber view demonstrating severe left atrial and left ventricular dilation. LA – left atrium, LV – left ventricle. **B **Apical four-chamber view demonstrating severe mitral and tricuspid regurgitation (arrows)

**Fig. 3:**
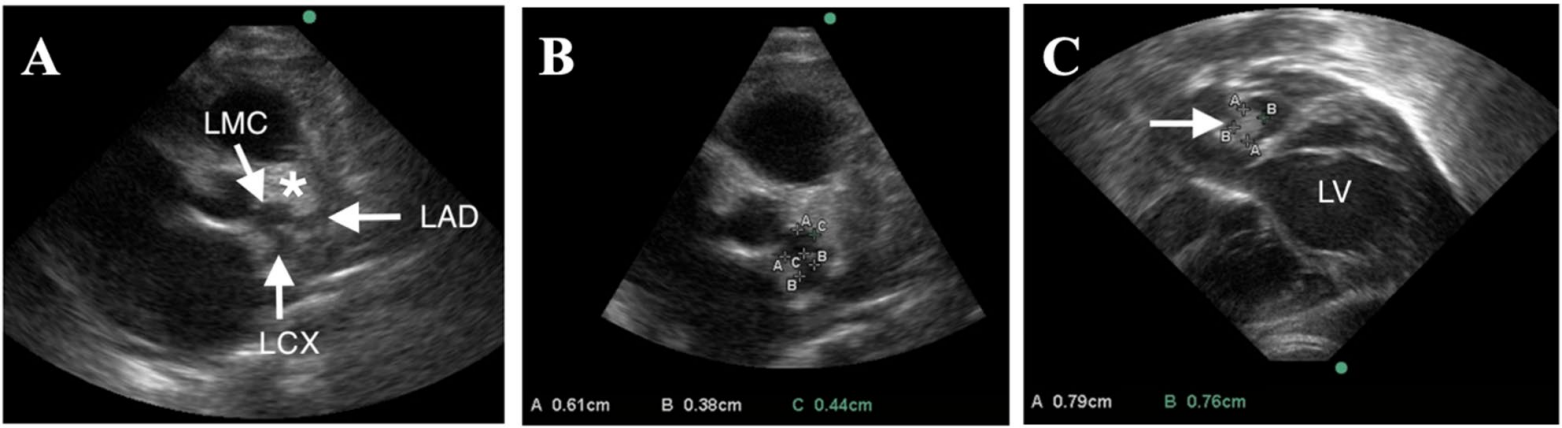
Transthoracic echocardiography demonstrating giant coronary artery aneurysm with intraluminal thrombus. **A **Parasternal short-axis view demonstrating marked dilation of the left coronary system. The left main coronary artery (LMC), left anterior descending artery (LAD), and left circumflex artery (LCX) are indicated by arrows. An echogenic intraluminal thrombus within the aneurysmal segment is marked with an asterisk (*). **B **Measurement of functional luminal diameters of the LMC, LAD, and LCX. **C **Subcostal view illustrating a large aneurysmal coronary segment with echogenic intraluminal thrombus (arrow) adjacent to the left ventricle (LV).

Chest radiography demonstrated marked cardiomegaly with a cardiothoracic ratio of approximately 0.69, accompanied by pulmonary edema (Fig. 1B), representing a dramatic progression from a previously normal chest radiograph obtained during her initial admission at four months of age (Fig. 1A). Transthoracic echocardiography revealed significant left heart dilation with the following measurements: left atrium 2.5 cm (Z-score + 2.33), left ventricular end-diastolic dimension 3.2 cm (Z-score + 2.65), and left ventricular end-systolic dimension 3.0 cm (Z-score + 8.14). Left ventricular systolic function was severely depressed with an estimated ejection fraction of 13%. Severe mitral and tricuspid regurgitation were present together with moderate pulmonary hypertension (Fig. 2A, B). Marked coronary artery dilation was also noted: the functional luminal diameter of the left main coronary artery (LMC) measured 6.1 mm (Z-score + 11.4), the left anterior descending artery (LAD) 4.0 mm (Z-score + 10.7), and the left circumflex artery (LCX) 3.8 mm (Fig. 3A, B). Z-scores for both cardiac chamber dimensions and coronary arteries were calculated using the Boston Children’s Hospital Z-score calculator [9]. Two intraluminal thrombi were visualized within a giant left proximal coronary aneurysm, measuring 8 × 9 mm and 14 × 10 mm. A bovine aortic arch variant was also noted, with a common origin of the brachiocephalic and left common carotid arteries. 

Repeat laboratory investigations demonstrated leukocytosis (WBC of 15.2 × 10^9^/L, 48% lymphocytes), worsening anemia (hemoglobin of 8.2 g/dL), and a platelet count of 357 × 10^9^/L. Given the presence of giant coronary artery aneurysms with intraluminal thrombus and severe left ventricular systolic dysfunction, the clinical picture was consistent with advanced KD complicated by cardiogenic shock. Management decisions focused on stabilization and prevention of further thrombotic complications. The patient was therefore treated with inotropic support using dopamine together with dual antiplatelet therapy (aspirin and clopidogrel) and anticoagulation with enoxaparin. Despite aggressive supportive management, she remained hypotensive and progressed to refractory cardiogenic shock, ultimately resulting in death within 12 h of ICU admission.

A retrospective review of her medical history revealed a previous admission at four months of age for nine days of persistent fever without an identifiable source. She was empirically treated with intravenous antibiotics and an antimalarial despite a negative malarial blood film. Laboratory findings during that admission showed leukocytosis (WBC 18.1 × 10^9^/L, neutrophils 59.3%), anemia (hemoglobin 8.9 g/dL), microscopic hematuria, pyuria, and markedly elevated inflammatory markers (C-reactive protein 178 mg/L). The platelet count was within the normal range (293 × 10^9^/L). The fever gradually resolved during hospitalization, and she was discharged without a definitive diagnosis.

Ten days following discharge, she presented again with seizures. Neuro-imaging with transfontanelle ultrasound and laboratory evaluations were normal. She was diagnosed with epilepsy and commenced on antiepileptic therapy, with no further seizures reported. The chronological clinical course of the patient is summarized in Table 1. 

**Table 1:** Timeline of clinical events, investigations, and management

Age / Time	Clinical Events	Investigations	Management / Outcome
4 months (Day 1–9)	Persistent fever without clear source	CBC: WBC 18.1 × 10^9^/L, Hb 8.9 g/dL; CRP 178 mg/L; sterile pyuria; normal chest radiograph	Empirical IV antibiotics and antimalarial treatment
Day 8 of illness	Echocardiography performed	Reported as normal except for bovine aortic arch	No KD-specific treatment given
10 days after discharge	Seizure episode	Neuroimaging and labs normal	Diagnosed with epilepsy, started antiepileptic therapy
6 months (2 months later)	Grunting, respiratory distress, vomiting	CBC: leukocytosis; abdominal ultrasound showing pleural effusion and dilated cardiac chambers	Referred to ICU
ICU admission	Cardiogenic shock	Echocardiography showing giant coronary aneurysm, thrombus, severe LV dysfunction	Dopamine, aspirin, clopidogrel, enoxaparin
12 h after ICU admission	Refractory cardiogenic shock		Death

*Abbreviations:* *CBC *complete blood count, *CRP *C-reactive protein, *Hb *hemoglobin, *IV *intravenous, *ICU *intensive care unit, *KD* Kawasaki disease, *LV* left ventricle, *WBC *white blood count

### Diagnostic reasoning

At the time of the initial febrile illness, infectious etiologies were considered most likely given the high prevalence of infectious diseases in the local setting. The patient was therefore empirically treated for presumed bacterial infection and malaria despite negative malarial blood film. KD was not initially suspected because the patient lacked the classic clinical features of the disease.

In retrospect, the presence of prolonged fever, markedly elevated inflammatory markers, anemia, leukocytosis, and sterile pyuria fulfilled the supplementary laboratory criteria included in the diagnostic algorithm for incomplete KD recommended by the American Heart Association (AHA) [10]. Earlier recognition of these findings could have prompted echocardiographic surveillance and consideration of intravenous immunoglobulin therapy during the initial hospitalization.

## Discussion

Infants are at particularly high risk of developing coronary artery aneurysm (CAA) following KD, with reported prevalence rates as high as 68% in infants younger than six months [11]. This age group is also more likely to present with incomplete or atypical disease, contributing to delayed or missed diagnosis and increased morbidity and mortality.

During the initial admission at four months of age, the patient had nine days of unexplained fever together with markedly elevated inflammatory markers (CRP 178 mg/L). Supplemental laboratory findings included anemia (hemoglobin 8.9 g/dL), leukocytosis (WBC 18.1 × 10^9^/L), and sterile pyuria, all of which are supportive laboratory criteria included in the incomplete KD diagnostic algorithm recommended by the AHA. In infants younger than six months with prolonged unexplained fever and elevated inflammatory markers, the AHA recommends evaluation for incomplete KD and performance of echocardiography even in the absence of classical clinical features [10]. This recommendation reflects the particularly high risk of coronary artery complications in this age group.

In our patient, echocardiography was performed on the eighth day of illness during her initial febrile admission to evaluate possible cardiac causes of persistent fever, including myocarditis or infective endocarditis, rather than specifically to assess for coronary artery involvement related to KD. The echocardiography result was described as unremarkable aside from a bovine aortic arch variant. As KD was not suspected clinically, scheduled follow-up echocardiography was not preformed.

In retrospect, despite the initial normal echocardiography, her findings fulfilled the criteria that should have prompted consideration of incomplete KD and initiation of treatment during the initial hospitalization [12]. The AHA also recommends a repeat echocardiogram within 1 to 2 weeks after discharge for patients without coronary artery involvement during hospitalization, as a small number of patients will develop coronary enlargement within a week or two after discharge, and earlier detection allows prompt institution of adjunctive anti-inflammatory therapy [1].

Notably, thrombocytosis was absent at the initial presentation, a finding that has been described in early or severe cases of KD in young infants [2].

Incomplete KD has been characterized by prolonged fever, young age at onset, frequent BCG (Bacille Calmette-Guerin) scar reaction, higher rates of coronary artery involvement, and enhanced immune tolerance to immunoglobulin [13]. In the absence of typical clinical features, KD was not suspected in this case, and treatment was directed toward presumed infectious causes. In settings where epidemiological data on KD are scarce, including Ethiopia, there is often a tendency to discount KD as a diagnostic possibility and prioritize more commonly encountered infections in febrile infants. This case underscores the critical importance of maintaining a high index of suspicion for KD and adhering to established diagnostic algorithms while evaluating febrile children, even in resource-limited settings; lest we overlook a critical diagnosis hidden among more common conditions.

An additional point of interest is the episode of seizures and subsequent diagnosis of epilepsy. Although neuroimaging was normal, neurological involvement has been described in incomplete KD, including seizures, aseptic meningitis, irritability, and facial nerve palsy [14]. A nationwide cohort study from Taiwan demonstrated an association between KD and subsequent childhood epilepsy, with higher risk observed in females and children under five years of age [15]. It is therefore possible that the neurological episode represented an inflammatory manifestation related to KD; however, given the limited neurological evaluation performed, this association remains speculative.

Finally, the presence of a bovine aortic arch in this patient, while likely incidental, has been described in association with altered vascular flow dynamics and other vascular anomalies [16]. Bovine aortic arch is the most common aortic arch branching variant in humans, occurring in approximately 27% of the population [17].

## Conclusion

This case highlights the fatal consequences of missed incomplete KD in a young infant. Clinicians must remain vigilant when evaluating infants with prolonged unexplained fever and elevated inflammatory markers, even in the absence of classic clinical features of KD. Early recognition and strict adherence to established diagnostic and treatment algorithms are essential to prevent irreversible coronary damage and death. Increased awareness of KD in low-resource settings is needed to avoid missing this potentially treatable yet devastating condition.

## Supplementary Information

Supplementary Material 1.

## Data Availability

All data generated or analyzed during this study are included in this published article.

## Abbreviations

AHA: American Heart Association
BCG: Bacille Calmette–Guerin
BiPAP: Bilevel positive airway pressure
CAA: Coronary artery aneurysm
CBC: Complete blood count
ICU: Intensive care unit
IV: Intravenous
KD: Kawasaki disease
LA: Left atrium
LAD: Left anterior descending artery
LCX: Left circumflex artery
LMC: Left main coronary artery
LV: Left ventricle
WBC: White blood cell
