# Supplementary material for: Fatal incomplete Kawasaki disease in a six-month-old infant from Ethiopia: a case report: Author
Source: BMC Pediatr. 2026 Mar 19;26:383. doi: 10.1186/s12887-026-06753-2 (PMC13122887; doi:10.1186/s12887-026-06753-2)
Supplement: Supplementary file 1 — Supplementary Material 1. [file 12887_2026_6753_MOESM1_ESM.pdf]

CARE
case report guidelines

CARE Checklist of information to include when writing a case report

(cc) BY-NC-ND

Topic	Item	Checklist item description	Reported on Line
Title	1	The diagnosis or intervention of primary focus followed by the words "case report"	1
Key Words	2	2 to 5 key words that identify diagnoses or interventions in this case report, including "case report"	45, 46
Abstract (no references)	3a	Introduction: What is unique about this case and what does it add to the scientific literature?	26, 27
	3b	Main symptoms and/or important clinical findings	29 - 33
	3c	The main diagnoses, therapeutic interventions, and outcomes	33 - 35
	3d	Conclusion—What is the main "take-away" lesson(s) from this case?	37 - 42
Introduction	4	One or two paragraphs summarizing why this case is unique (**may include references**)	60 - 65
Patient Information	5a	De-identified patient specific information.	67 - 147
	5b	Primary concerns and symptoms of the patient.	68 - 70, 77, 82
	5c	Medical, family, and psycho-social history including relevant genetic information	134 - 141
	5d	Relevant past interventions with outcomes	135 - 136, 139 - 140
Clinical Findings	6	Describe significant physical examination (PE) and important clinical findings.	85 - 87
Timeline	7	Historical and current information from this episode of care organized as a timeline	147
Diagnostic Assessment	8a	Diagnostic testing (such as PE, laboratory testing, imaging, surveys).	94 - 126
	8b	Diagnostic challenges (such as access to testing, financial, or cultural)	190 - 194
	8c	Diagnosis (including other diagnoses considered)	152 - 160
	8d	Prognosis (such as staging in oncology) where applicable	Not applicable
Therapeutic Intervention	9a	Types of therapeutic intervention (such as pharmacologic, surgical, preventive, self-care)	129 - 132
	9b	Administration of therapeutic intervention (such as dosage, strength, duration)	130 - 132
	9c	Changes in therapeutic intervention (with rationale)	Not applicable
Follow-up and Outcomes	10a	Clinician and patient-assessed outcomes (if available)	132, 133
	10b	Important follow-up diagnostic and other test results	94 - 126
	10c	Intervention adherence and tolerability (How was this assessed?)	Not applicable
	10d	Adverse and unanticipated events	132 - 133
Discussion	11a	A scientific discussion of the strengths AND limitations associated with this case report	191 - 197
	11b	Discussion of the relevant medical literature **with references**.	161 - 209
	11c	The scientific rationale for any conclusions (including assessment of possible causes)	170 - 174, 180 - 185
	11d	The primary "take-away" lessons of this case report (without references) in a one paragraph conclusion	211 - 216
Patient Perspective	12	The patient should share their perspective in one to two paragraphs on the treatment(s) they received	Not applicable
Informed Consent	13	Did the patient give informed consent? Please provide if requested	Yes ☒ No ☐
